# The impact of intimate partner violence on PrEP adherence among U.S. Cisgender women at risk for HIV

**DOI:** 10.1186/s12889-024-18946-4

**Published:** 2024-05-31

**Authors:** Katherine M. Anderson, Jill Blumenthal, Sonia Jain, Xiaoying Sun, K. Rivet Amico, Raphael Landovitz, Christine M. Zachek, Sheldon Morris, David J. Moore, Jamila K. Stockman

**Affiliations:** 1 Division of Infectious Diseases and Global Public Health, Department of Medicine, School of Medicine, University of California, 9500 Gilman Dr., La Jolla, San Diego, CA 92093-0507 USA; 2 Department of Behavioral, Social, and Health Education Sciences, Rollins School of Public Health, Emory University, 1518 Clifton Rd, Atlanta, GA 30322 USA; 3 Biostatistics Research Center, Herbert Wertheim School of Public Health and Human Longevity Science, University of California, 9500 Gilman Dr., La Jolla, San Diego, CA 92093-0725 USA; 4 Department of Health Behavior and Health Education, School of Public Health, University of Michigan, Ann Arbor, MI 48109 USA; 5 David Geffen School of Medicine, UCLA Center for Clinical AIDS Research & Education, University of California Los Angeles, Los Angeles, CA 90024 USA; 6 Department of Obstetrics, Gynecology & Reproductive Sciences, University of California San Diego Health, 9300 Campus Point Drive, La Jolla, San Diego, CA 92037 USA; 7 Department of Psychiatry, University of California San Diego, San Diego, CA 92103 USA

**Keywords:** Intimate Partner violence, Pre-exposure Prophylaxis for HIV Prevention, Adherence, Violence

## Abstract

**Background:**

Cisgender women account for 1 in 5 new HIV infections in the United States, yet remain under-engaged in HIV prevention. Women experiencing violence face risk for HIV due to biological and behavioral mechanisms, and barriers to prevention, such as challenges to Pre-Exposure Prophylaxis for HIV Prevention (PrEP) adherence. In this analysis, we aim to characterize intimate partner violence (IPV) among cisgender heterosexual women enrolled in a PrEP demonstration project and assess the associations with PrEP adherence.

**Methods:**

Adherence Enhancement Guided by Individualized Texting and Drug Levels (AEGiS) was a 48-week single-arm open-label study of PrEP adherence in HIV-negative cisgender women in Southern California (*N* = 130) offered daily tenofovir disoproxil fumarate/emtricitabine (TDF/FTC). From 6/2016 to 10/2018, women completed a survey reporting HIV risk behavior and experiences of any IPV (past 90-days) and IPV sub-types (past-year, lifetime) and biological testing for HIV/STIs at baseline, and concentrations of tenofovir-diphosphate (TFV-DP) in dried blood spots at weeks 4, 12, 24, 36, and 48. Outcomes were TFV-DP concentrations consistent with ≥ 4 or ≥ 6 doses/week at one or multiple visits. Multivariable logistic regression models were conducted to examine associations.

**Results:**

Past-90-day IPV was reported by 34.4% of participants, and past-year and lifetime subtypes reported by 11.5-41.5%, and 21.5-52.3%, respectively. Women who engaged in sex work and Black women were significantly more likely to report IPV than others. Lifetime physical IPV was negatively associated with adherence at ≥ 4 doses/week at ≥ 3 of 5 visits, while other relationships with any IPV and IPV sub-types were variable.

**Conclusion:**

IPV is an indication for PrEP and important indicator of HIV risk; our findings suggest that physical IPV may also negatively impact long-term PrEP adherence.

**Clinical Trials Registration:**

NCT02584140 (ClinicalTrials.gov), registered 15/10/2015.

## Background

Cisgender women in the United States account for approximately 20% of new HIV infections [1] yet are significantly underrepresented in HIV prevention efforts. Furthermore, new HIV infections among cisgender women disproportionately occur among Black and Latina women [1]. From 2010 to 2015, Black and Latina women were 15.1 and 3.1 times as likely to be diagnosed with HIV as their White counterparts [1], while Black and African American-identifying cisgender women alone account for approximately 60% of all women living with HIV in the United States [1]. Once diagnosed, cisgender women are less likely to reach viral suppression compared to cisgender men, as are Latina and Black cisgender women compared to White cisgender women, indicating a need for prioritization of this population [2]. Therefore, there is significant need for HIV prevention efforts for cisgender women, and cisgender women of color specifically.

Violence and HIV act as mutually reinforcing epidemics, or syndemics, among cisgender women [3–7]. Intimate partner violence (IPV)- physical, sexual, and/or psychological violence, or stalking, against a woman perpetrated by a current or former sexual or romantic partner [8]- is among the most pervasive types of violence experienced by women in the United States [8], and both men and women survivors of IPV may have increased risk of HIV acquisition from sexual risk behavior, such as condomless sex and having multiple concurrent sex partners [9, 10]. Women in violent relationships are less likely to refuse sex or report condom use during sex, while perpetrators of IPV are more likely to engage in behaviors associated with HIV risk external to their relationship-based sexual activity, and are more likely to refuse to use a condom [11]. Poverty, race/ethnicity, and sexual and gender identity may increase exposure to violence [8, 12, 13], potentially compounding HIV risk. Further, adverse mental health outcomes associated with experiences of violence and trauma [14] may limit the ability to negotiate safe sex practices [3], or lead to substance use [15] and risky sexual behaviors [16]. Biological mechanisms linking experiences of violence to increased HIV susceptibility including dysregulation of the hypothalamic-pituitary-adrenal axis with implications for immune functioning, and disruption of the cervicovaginal epithelium through sexual violence, have been identified among cisgender women, though the mechanisms through which increased susceptibility occurs are not thoroughly understood [17, 18].

Cisgender women have few options for HIV prevention that can be adopted and implemented without partner knowledge or consent [11, 19]. Pre-exposure prophylaxis (PrEP) is a biomedical strategy for HIV prevention that is self-controlled and highly effective when used as recommended [20]. Oral PrEP is considered usable without partner knowledge, consent, or involvement [19, 21], though women experiencing IPV may face additional challenges to usage [6]. Oral PrEP may also offer an opportunity for cisgender women at risk for HIV and experiencing violence to have consistent engagement with health care services [11, 19]. In 2015 it was estimated that up to 200,000 cisgender women had indication for PrEP [22], though many more may benefit from PrEP under more recent Centers for Disease Control (CDC) criteria that indicate any women sexually active in the past six months who have inconsistent or no condom use are indicated for PrEP [23]. Yet, as of 2016, 93% of PrEP users were male, and men use PrEP at a rate 14 times higher than that of women, despite HIV infection rates being 4.7 times higher among males [1, 24]. Racial and ethnic disparities present in HIV prevalence are echoed in PrEP uptake; in a national sample of 1,146 female PrEP users, only 26% were Black, less than 20% were Hispanic or Latina, and almost 50% were White [25].

According to a recent systematic review, 51-97% of cisgender women express willingness to use oral PrEP at time of presentation to healthcare services [26], and use of oral PrEP is considered acceptable to cisgender women, including among women experiencing IPV [11, 27]. Yet, even among cisgender women who initiate PrEP, adherence remains a challenge [28]. Cisgender women are more likely to discontinue PrEP than men [29], and face unique barriers to PrEP uptake and retention, such as medical mistrust, stigma, low perceived HIV risk, and previous negative medical experiences, particularly among women of color [19, 26, 30, 31]. For women in violent relationships, lack of partner support for PrEP may act as a barrier to adherence [19], and violence could be exacerbated if a sexual partner discovers PrEP use [11, 32]. Among women and men experiencing IPV in international settings, almost one-quarter reported interruptions to PrEP use, and these individuals were 2.6 times more likely to experience interruptions to PrEP use than those not experiencing IPV [33]. However, little literature exists on IPV typology, including physical, sexual, psychological, and injurious IPV, and their impact on PrEP adherence among cisgender women at risk for HIV.

The current analysis aims to characterize IPV among cisgender heterosexual women seeking PrEP and to assess the association of IPV with PrEP adherence in an open-label clinical trial of PrEP adherence support strategy among cisgender women at risk for HIV.

## Methods

### Participant recruitment and enrollment

Adherence Enhancement Guided by Individualized Texting and Drug Levels (AEGiS) was a 48-week single-arm open-label PrEP demonstration study to estimate PrEP adherence, retention, and persistence among cisgender women at risk for HIV taking once daily tenofovir disoproxil fumarate/emtricitabine (TDF/FTC). The study was registered on ClinicalTrials.gov (NCT02584140) on 15/10/2015. As reported in detail elsewhere [34], participants were enrolled between June 2016 and October 2018 at five Southern California study sites, four in Los Angeles and one in San Diego [34]. Criteria for risk included having a partner living with HIV for more than four weeks, engaging in sex in exchange for money, goods or services, having taken post-exposure prophylaxis for HIV (PEP) within the last year, having a bacterial sexually transmitted infection (STI) in the last 6 months, or having a partner of unknown HIV status with known HIV risk behaviors. Women were also required to be age 18 or older, speak English or Spanish, test negative for HIV by 4th generation antigen/antibody assay or antibody assay plus HIV nucleic acid test, and have creatinine clearance > 60 ml/minute [35]. Women interested in participating in the 48-week trial were identified through flyers, advertisements, and care providers at HIV testing sites, HIV clinics, community organizations, and women’s health clinics. Participants provided informed consent prior to study screening, then participated in study visits at weeks 0, 4, 12, 24, 36, and 48. TDF/FTC was provided to all participants at no cost at baseline and weeks 4, 12, 24 and 36. A self-administered computer assisted survey instrument (CASI) was used to assess baseline demographics, violence, and HIV risk behaviors as well as longitudinal assessments of HIV risk and medication use behavior. Additional details of the study protocol have been previously described [34]. Participants were compensated $10 at screening, and $50 at each completed study visit for their time and travel.

### Measures

Previous work with the sample data identified three HIV risk groups according to sexual HIV risk behaviors: (1) Being in a serodiscordant partnership; (2) engaging in sex work and (3) having partner(s) of unknown HIV status with known HIV risk behaviors [34]. Categories were mutually exclusive with participants placed in the highest risk category applicable to them, with serodiscordant relationships being highest risk, followed by sex work, followed by partner(s) of unknown HIV status with known risk behavior. CASI questions included demographic characteristics and experiences of violence, including IPV (items from Revised Conflicts Tactics Scale, CTS) [36]. Respondents indicated frequency of experience of each IPV item within the past year, that they did not experience it in the past year but had previously in their life, or that they have never experienced it. IPV was dichotomized into yes/no for lifetime and yes/no for past year experience of each subtype, wherein endorsement of any of the items within each subtype was classified as having experienced that type of IPV as follows: injurious IPV, physical IPV, psychological IPV, and sexual IPV [36] Past 90-day IPV was measured using a five-point Likert-scale capturing frequency of the following events perpetrated by an intimate partner: being threatened with a weapon, being beat you so badly that you had to seek medical help, being forced to have sex, or your partner having no respect for your feelings. Responses were dichotomized into any or no experience of past 90-day IPV. Experiences of IPV used in the analysis were reported at baseline and are relative to date of study enrollment.

TFV-DP concentrations were used to assess PrEP adherence, measured using a validated liquid chromatography–tandem mass spectrometry assay at weeks 4, 12, 24, 36, and 48 [37]. For any given visit, TFV-DP concentrations  ≥ 700 fmol/punch was defined as consistent with ≥ 4 doses per week, and TFV-DP concentration ≥ 1050 fmol/punch was defined as consistent with ≥ 6 doses per week (over the prior 1–2 months at steady state) [38]. Composite adherence outcomes derived from multiple study visits included: (1) adherence at ≥ 4 doses per week at one or more study visits attended, (2) adherence at ≥ 6 doses per week at one or more study visits attended, and (3) adherence at ≥ 4 doses/week at three or more study visits. Adherence at ≥ 6 doses/week at 3 or more study visits was computed, but not presented due to low frequency of the outcome. Participants lost to follow-up or with missed study visits were counted as non-adherent at that study visit and/or remaining missed study visits.

### Statistical analysis

We performed statistical analyses in R, version 4.1.2 (R Core Team, 2013). Descriptive analyses were used to summarize and compare IPV exposure by demographic characteristics and HIV risk groups. Group comparisons used Fisher’s exact test for the categorical variables, as small sample size led to a sub-sample of less than five in some categories. Cross-tabulation and Fisher’s exact test was used to assess the bivariate association between each IPV exposure and each PrEP composite adherence outcome. We also performed multivariable logistic regression models to study the association of each IPV exposure with each composite adherence outcome except “consistent ≥ 6 doses,” due to small categorical subsamples. Regressions were adjusted for theoretically-based covariates including continuous age, race (Black/non-Black, with the latter inclusive of White, Asian, Native American, Pacific Islander, and Other), ethnicity (Hispanic/non-Hispanic), education (High School or Less/More than High School), monthly income (<$2,000/≥$2,000), HIV risk group (serodiscordant relationship/sex worker/partner(s) of unknown HIV status with known HIV risk behaviors), and study site (LA/SD).

### Ethical approval

The research protocol was approved by the relevant Institutional Review Boards at University of California Los Angeles, Harbor-UCLA Medical Center, University of Southern California, and University of California San Diego. The study was registered at clinicaltrials.gov (NCT02584140).

## Results

### Participant characteristics

One-hundred and sixty-seven (167) women completed the screening survey, of whom 130 were eligible and completed a baseline study visit with available IPV data. At week 4, 91% (*n* = 118) participants were retained, followed by 84% (*n* = 109) at week 12, 75% (*n* = 97) at week 24, and 63% (*n* = 82) at week 48. Of 48 participants who did not complete a week 48 visit, 29% (*n* = 14) requested to withdraw; the remainder (*n* = 34) were lost to follow-up [34].

Of enrolled participants at baseline, 46.2% were in a serodiscordant relationship, 14.6% engaged in sex work, and 39.2% had partners with unknown HIV status and known HIV risk behaviors (Table 1). Women had a mean age of 40 years old (SD = 11, Range: 19–67) and were primarily Black (34.1%); 1.5% identified as Hispanic. Having greater than high school education was reported by 54.6%, and 54.6% had a monthly income less than $2,000. Approximately one-third of participants reported experiencing any type of IPV within the past 90 days. Injurious IPV was reported by 34.4% of women in the past year and by 21.5% in their lifetime (Tables 1 and 2); 11.5% and 30.0% of women reported physical IPV in the past year and their lifetime, respectively; psychological IPV was reported by 41.5% of women in the past year and 52.3% in their lifetime; and, sexual IPV was reported by 23.1% and 32.3% in the past year and in their lifetime.

**Table 1:** Demographic characteristics of Cisgender women at risk for HIV in Southern California, 2016–2018, and prevalence of past 90-day and past-year intimate partner violence (IPV) (*N* = 130)

	Total Sample*	Past 90-day IPV ^		Past Year Injurious IPV^		Past Year Physical IPV^		Past Year Psychological^		Past Year Sexual IPV^	
	*N* (%)	*n* (%)	*p*	*n* (%)	*p*	*n* (%)	*p*	*n* (%)	*p*	*n* (%)	*p*
Overall	*N* = 130	45 (34.4)	--	15 (11.5)	--	22 (16.9)	--	54 (41.5)	--	30 (23.1)	--
Age											
< 40	65 (50.0)	26 (40.0)	0.19	6 (9.2)	0.58	11 (16.9)	> 0.99	26 (40.0)	0.86	17 (26.2)	0.53
40+	65 (50.0)	18 (27.7)		9 (13.9)		11 (16.9)		28 (43.1)		13 (20.0)	
Race/Ethnicity											
Hispanic	41 (31.5)	11 (26.8)	0.32	2 (4.9)	0.14	5 (12.2)	0.45	13 (31.7)	0.13	8 (19.5)	0.66
Non-Hispanic	88 (67.7)	33 (37.5)		13 (14.8)		17 (19.3)		41 (46.6)		22 (25.0)	
Black	56 (34.1)	24 (42.9)	0.06	10 (17.9)	0.06	13 (23.2)	0.11	28 (50.0)	0.11	17 (30.4)	0.09
Non-Black	74 (56.9)	20 (27.0)		5 (6.8)		9 (12.2)		26 (35.1)		13 (17.6)	
Enrollment Site											
LA	90 (69.2)	30 (33.3)	0.84	**14 (15.6)**	**0.04**	19 (21.1)	0.08	41 (45.6)	0.18	22 (24.5)	0.66
San Diego	40 (30.8)	14 (35.0)		**1 (2.5)**		3 (7.5)		13 (32.5)		8 (20.0)	
Formal Education											
High School or Less	59 (45.4)	20 (33.9)	> 0.99	9 (15.2)	0.28	10 (17.0)	> 0.99	24 (40.7)	> 0.99	13 (22.1)	0.84
More than High School	71 (54.6)	24 (33.8)		6 (8.5)		12 (16.9)		30 (42.3)		17 (23.9)	
Monthly Income											
<$2,000	71 (54.6)	26 (36.6)	0.27	**13 (18.3)**	**0.01**	17 (23.9)	0.08	32 (45.1)	0.34	17 (23.9)	0.67
≥$2,000	34 (26.2)	13 (38.2)		**0 (0.0)**		3 (8.8)		15 (44.1)		9 (26.5)	
Unknown	25 (19.2)	5 (20.0)		**2 (8.0)**		2 (8.0)		7 (28.0)		4 (16.0)	
HIV Risk Factors											
Sero-discordant	60 (46.2)	16 (26.7)	0.20	4 (6.7**)**	0.08	**6 (10.0)**	**< 0.01**	25 (41.7)	0.51	10 (16.7)	0.08
Sex Work	19 (14.6)	6 (31.6)		5 (26.3)		**8 (42.1)**		10 (52.6)		8 (42.1)	
Partner with Unknown Status	51 (39.2)	22 (43.1)		6 (11.8)		**8 (15.7)**		19 (37.3)		12 (23.5)	

*Column percent, ^Row percent

Notes: Percentages may not add up to 100 due to missing data. Bolded values indicate significance at a level of *p* ≤ 0.05

Bivariate associations between demographics and past 90-day, past-year, and lifetime experiences of IPV are presented in Tables 1 and 2. Black cisgender women, compared to women identifying as any other race, reported a higher proportion of past 90-day IPV (42.9% vs. 27.0%, *p* = 0.06), past-year injurious IPV (17.9% vs. 6.8%, *p* = 0.06), past-year sexual violence (30.4% vs. 17.6%, *p* = 0.09), and lifetime sexual IPV (42.9% vs. 24.3%, *p* = 0.04). Cisgender women with low income (<$2,000/month) reported higher past-year injurious IPV. Across all categories of lifetime and past-year IPV, a higher proportion of women who engaged in sex work reported experiences of IPV than cisgender women in other risk groups, with significant differences between risk groups for past-year and lifetime physical IPV, lifetime injurious IPV, lifetime sexual IPV, and non-significant differences for past-year injurious IPV and past-year sexual IPV (both *p* = 0.08).

**Table 2:** Prevalence of lifetime intimate partner violence (IPV) by demographic characteristics among Cisgender women at risk for HIV in Southern California, 2016–2018 (*N* = 130)

	Lifetime Injurious IPV		Lifetime Physical IPV		Lifetime Psychological IPV		Lifetime Sexual IPV	
	*n* (%)	*p*	*n* (%)	*p*	*n* (%)	*p*	*n* (%)	*p*
Overall	28 (21.5)		39 (30.0)		68 (52.3)		42 (32.3)	
Age								
40+	14 (21.5)	> 0.99	19 (29.2)	> 0.99	34 (52.3)	> 0.99	24 (36.9)	0.35
40+	14 (21.5)		20 (30.8)		34 (52.3)		18 (27.7)	
Race/Ethnicity								
Hispanic	5 (12.2)	0.12	8 (19.5)	0.1	19 (46.3)	0.35	9 (22.0)	0.11
Non-Hispanic	23 (26.1)		31 (35.2)		49 (55.7)		33 (37.5)	
Black	15 (26.8)	0.28	21 (37.5)	0.12	34 (60.7)	0.11	**24 (42.9)**	**0.04**
Non-Black	13 (17.6)		18 (24.3)		34 (46.0)		**18 (24.3)**	
Enrollment Site								
LA	22 (24.4)	0.26	**33 (36.7)**	**0.01**	51 (56.7)	0.18	32 (35.6)	0.31
San Diego	6 (15.0)		**6 (15.0)**		17 (42.5)		10 (25.0)	
Formal Education								
High School or Less	13 (22.0)	> 0.99	18 (30.5)	> 0.99	32 (54.2)	0.73	19 (32.2)	> 0.99
More than High School	15 (21.1)		21 (29.6)		36 (50.7)		23 (32.4)	
Monthly Income								
<$2,000	17 (23.9)	0.48	25 (35.2)	0.33	38 (53.5)	0.63	23 (32.4)	0.53
$2,000	8 (23.5)		9 (26.5)		19 (55.9)		13 (38.2)	
Unknown	3 (12.0)		5 (20.0)		11 (44.0)		6 (24.0)	
HIV Risk Factors								
Serodiscordant	**9 (15.0)**	**0.02**	**12 (20.0)**	**< 0.01**	29 (48.3)	0.14	**13 (21.7)**	**< 0.01**
Sex Work	**9 (47.4)**		**13 (68.4)**		14 (73.7)		**12 (63.2)**	
Partner with Unknown Status	**10 (19.6)**		**14 (27.5)**		25 (49.0)		**17 (33.3)**	

Notes: Percentages may not add up to 100 due to missing data. Bolded values indicate significance at a level of *p* ≤ 0.05

Table 3 shows the bivariate associations between past 90-day, past-year, and lifetime IPV and each of the four adherence outcomes (TFV-DP blood concentrations consistent with ≥ 4 and ≥ 6 at one or more visits, or ≥ 4 and ≥ 6 doses at three or more study visits). There were no significant associations between past 90-day, past-year, or lifetime experiences of IPV and achieving adherence consistent with ≥ 4 doses of PrEP per week at one or more visits in the trial. However, IPV experiences were negatively associated with adherence consistent with ≥ 6 doses of PrEP per week at one of more visits in the trial among those who experienced past-year physical IPV (27.3% vs. 53.7%, *p* = 0.03), lifetime physical IPV (30.8% vs. 57.1%, *p* < 0.01), lifetime psychological IPV (41.2% vs. 58.1%, *p* = 0.08) and lifetime sexual IPV (33.3% vs. 56.8%, *p* = 0.02), compared to those who did not experience each type of IPV. Of IPV experiences, only lifetime physical violence was associated with not being adherent at a level consistent with ≥ 4 doses per week at three or more study visits (10.3% vs. 35.2%, *p* < 0.01), or ≥ 6 doses per week at three or more study visits (0.0% vs. 15.4%, *p* = 0.01).

**Table 3:** Bivariate associations of intimate partner violence and pre-exposure prophylaxis for HIV prevention (PrEP) adherence among Cisgender women at risk for HIV in Southern California, 2016–2018 (*N* = 130)

		*n*	Ever ≥ 4 doses		Ever ≥ 6 doses		Consistent ≥ 4 doses		Consistent ≥ 6 doses	
			*N* (%)	*p*	*N* (%)	*p*	*N* (%)	*p*	*N* (%)	*p*
Past 90-Day IPV	*N*	86	60 (69.8)	0.24	47 (54.7)	0.10	27 (31.4)	0.22	11 (12.8)	0.38
	Y	44	26 (59.1)		17 (38.6)		9 (20.5)		3 (6.8)	
LT Injurious IPV	*N*	102	68 (66.7)	0.82	52 (51.0)	0.52	31 (30.9)	0.24	12 (11.8)	0.77
	Y	28	18 (64.2)		12 (42.9)		5 (17.9)		2 (7.1)	
PY Injurious IPV	*N*	115	78 (67.8)	0.38	59 (51.3)	0.27	34 (29.6)	0.23	13 (11.3)	> 0.99
	Y	15	8 (53.3)		5 (33.3)		2 (13.3)		1 (6.7)	
PY Physical IPV	*N*	108	73 (67.6)	0.47	**58 (53.7)**	**0.03**	33 (30.6)	0.12	14 (13.0)	0.13
	Y	22	13 (59.1)		**6 (27.3)**		3 (13.6)		0 (0.0)	
LT Physical IPV	*N*	91	63 (69.2)	0.31	**52 (57.1)**	**< 0.01**	**32 (35.2)**	**< 0.01**	**14 (15.4)**	**0.01**
	Y	39	23 (59.0)		**12 (30.8)**		**4 (10.3)**		**0 (0)**	
PY Psychological IPV	*N*	76	52 (68.4)	0.57	41 (54.0)	0.22	22 (29.0)	0.84	6 (7.9)	0.26
	Y	54	34 (63.0)		23 (42.6)		14 (25.9)		8 (14.8)	
LT Psychological IPV	*N*	62	43 (69.4)	0.58	36 (58.1)	0.08	20 (32.3)	0.33	6 (9.7)	0.78
	Y	68	43 (63.2)		28 (41.2)		16 (23.5)		8 (11.8)	
PY Sexual IPV	*N*	100	67 (67.0)	0.83	53 (53.0)	0.15	29 (29.0)	0.65	12 (12.0)	0.52
	Y	30	19 (63.3)		11 (36.7)		7 (23.3)		2 (6.7)	
LT Sexual IPV	*N*	88	60 (68.2)	0.55	**50 (56.8)**	**0.02**	28 (31.8)	0.15	12 (13.6)	0.22
	Y	42	26 (61.9)		**14 (33.3)**		8 (19.1)		2 (4.8)	

IPV = Intimate partner violence; LT = Lifetime; PY = Past year; N = No; Y = Yes

Notes: Percentages may not add up to 100 due to missing data. Bolded values indicate significance at a level of *p* ≤ 0.05

In adjusted regressions, no single type of IPV (Physical, Sexual Psychological, Injury) or recency of IPV (past 90-day, past-year) was statistically significantly associated with attending at least one study visit at which biological samples indicated PrEP adherence consistent with ≥ 4 or ≥ 6 doses/week (Table 4). However, lifetime physical IPV was associated with decreased odds of indication of adherence at ≥ 3 of 5 study visits at a level consistent with ≥ 4 dose/week (OR = 0.19, 95%CI: 0.05, 0.77, *p* = 0.02).

**Table 4:** Adjusted odds of pre-exposure prophylaxis for HIV prevention (PrEP) Adherence consistent with ≥ 4 and ≥ 6 doses/week* among Cisgender women at risk for HIV in Southern California, 2016–2018 (*N* = 130)

	Ever ≥ 4 doses		Ever ≥ 6 doses		Consistent ≥ 4	
Intimate Partner Violence Recency/Type	aOR	95% CI	aOR	95% CI	aOR	95% CI
Past 90d	0.660	0.294, 1.479	0.539	0.241, 1.206	0.659	0.244, 1.782
PY Physical	0.710	0.249, 2.025	0.458	0.152, 1.376	0.550	0.125, 2.427
PY Sexual	0.885	0.356, 2.203	0.617	0.248, 0.152	0.954	0.312, 2.912
PY Psychological	0.779	0.364, 1.754	0.734	0.342, 1.577	1.065	0.429, 2.644
PY Injurious	0.615	0.184, 2.048	0.773	0.226, 2.642	0.744	0.134, 4.125
LT Physical	0.659	0.270, 1.608	0.414	0.167, 1.030	**0.191**	**0.047, 0.768**
LT Sexual	0.824	0.351, 1.938	0.440	0.186, 1.039	0.592	0.202, 1.729
LT Psychological	0.802	0.365, 1.761	0.605	0.284, 1.288	0.809	0.22, 1.972
LT Injurious	0.984	0.382, 2.532	0.956	0.377, 2.428	0.557	0.163, 1.909
Any LT IPV	0.951	0.413, 2.190	0.819	0.368, 1.822	1.038	0.402, 2.680

*Regressions adjusted for age, race (Black/non-Black), ethnicity (Hispanic/non-Hispanic), education (> High school/≤High school), monthly income (≥$2,000/<$2,000/Unknown), study site (LA/SD), Risk group (Sex Work/Partner(s) of unknown status/serodiscordant relationship)

IPV = Intimate partner violence; LT = Lifetime; PY = Past year

## Discussion

We aimed to assess the association of IPV with PrEP adherence among cisgender women at risk for HIV enrolled in an open-label clinical trial of PrEP adherence support. Echoing previous findings [33], experiences of IPV were negatively associated with PrEP adherence in the AEGiS trial; however, the associations were variable, with physical IPV retaining the most consistent significance. This study is the first, to our knowledge, to describe the association between exposure to IPV subtypes and PrEP adherence. Despite significant associations at the bivariate level, in regression analyses TFV-DP concentration consistent with ≥ 4 or ≥ 6 doses/week at one of more visits in the trial was not associated with IPV. These findings imply that achieving adherence of at least 4 dose/week at any given health care visit is feasible for women experiencing IPV. However, women who had experienced lifetime physical IPV had lower odds of reaching TFV-DP blood levels consistent with ≥ 4 dose/week at ≥ 3 of 5 study visits. These findings suggest that women who have experienced lifetime physical IPV should be prioritized for interventions to promote PrEP adherence and are particularly in need of support for PrEP continuation at a protective level.

Due to the nature of IPV as a traumatic experience, special attention must be paid to the nature of PrEP clinical and support services provided to women experiencing IPV. Health care environments may unknowingly replicate circumstances of IPV (e.g. physical examinations, disempowerment) [39] triggering a re-experiencing of trauma and its physical and psychological sequalae, known as re-traumatization. Health care providers may be unaware of the trauma their patients have experienced [40], and therefore unable to incorporate practices that reduce the risk of re-traumatization, or trauma-informed care (TIC). While fear of re-traumatization may lead to health care avoidance [39], TIC is an evidence-based practice that, when incorporated into service delivery for survivors of trauma, can increase patient comfort and the acceptability of care [41] and potentially lead to improved engagement and retention in care [42]. Organizational-level implementation of TIC is vital to engaging populations most at risk for HIV in preventative care [43–45]; however, provider-level implementation may provide a stopgap while organizational capacity to implement TIC is built. Screening for IPV among women at risk for HIV can allow targeting of TIC capacity and resources, and integration of violence screening may leverage existing resources and systems [40], requiring less organizational overhead than adoption of TIC [40]. However, survivors may choose not to disclose their experiences, and universal application of TIC would benefit individuals at risk for HIV who have experienced non-IPV trauma that may similarly compromise retention in care and PrEP adherence.

Exposure to trauma, such as IPV, is associated with dysregulation of the stress and immune responses, decreasing the body’s ability to respond and prevent HIV infection [17, 18]. Violence-related stress may be further compounded by stress associated with minority identity, including Black race and sex work as a profession, marking these groups at particular risk for HIV. Further, sexual violence is associated with increased risk of HIV transmission, due to disruption of the cervicovaginal epithelium during rape [18]. Associations of experiences of violence and minority group membership with inconsistent PrEP adherence, therefore, are of particular concern; the same experiences that increase biological HIV risk for women upon exposure reduce the odds of adherence to PrEP. Black women in particular were more likely to have experienced lifetime violence in our sample and nationally, and they are systematically under-accessed for PrEP uptake. Yet, efforts to increase PrEP uptake alone are insufficient without prevention-effective adherence. Trauma-informed counseling on HIV prevention with the full range of options, such as through a model of shared decision-making, and with meaningful consideration for circumstances that may make adherence to an oral PrEP regimen difficult are vital. Discussing the various routes of PrEP administration is of particular importance with the recent approval of long-acting injectable cabotegravir for PrEP [46], and the likelihood of additional PrEP modalities for cisgender women in the coming years- which, like the injectable, may entail less frequent usage. Clinics and organizations serving cisgender women who are at risk for HIV, and particularly survivors of violence, should strengthen their capacity to support PrEP adherence and ensure comprehensive, trauma-informed counseling on HIV prevention modalities.

Our study has some limitations. Behavioral risk measures and IPV history were based on self-report and may be subject to recall bias or social desirability bias, though the high prevalence among our sample of reports of physical, sexual, injurious, and psychological IPV, experiences that are known to be underreported, suggests that such biases were of minimal effect. The composite variable used to measure past 90-day IPV only accounted for five behavioral-specific aspects of physical, sexual, and psychological IPV. It is unknown whether other violent behaviors were experienced by women, which could have resulted in an underestimate of the proportion reporting past 90-day IPV. The sample size for AEGiS was relatively small and analyses accounted for several potential confounders, limiting statistical power, but there was an adequate distribution of women across the variables of interest. Multiple theoretically-based covariates were adjusted for in regression analyses, which could lead to overfitting of the model, and assessing for effect modification may be an important next analytic step. All variables were selected based on established importance in the literature or behavioral health theory. Income and education were both included in the adjusted models and appear correlated; in sensitivity analyses where the income variable is removed from the models, the results are consistent. Both were retained in the model due to the established important of education and income independently in the literature. Additionally, there was loss to follow-up across study visits; however, these women were treated as non-adherent, reflecting more conservative estimates of PrEP adherence; this is consistent with other PrEP demonstration projects [34]. In the analysis phase, we chose not to adjust for multiple tests due to the small sample; given this, we acknowledge that while consistent with previous literature, it is possible that the findings are spurious. Although the trial allowed for a longitudinal assessment of IPV on PrEP adherence, this assessment was retrospective in nature and IPV exposure was relative to the baseline study visit. Therefore, the length of time between IPV and PrEP continuation increased at each study timepoint. Finally, participants in AEGiS were receiving active text message support for PrEP adherence, therefore levels of adherence may overestimate those of women in the general population not receiving support.

Despite these limitations, our study contributes to the dearth of research examining the role of IPV in PrEP adherence among cisgender at risk women, a population that has significantly lower PrEP initiation rates than men. Given that IPV was a central focus of this study, assessment of IPV using validated measures strengthened the validity of our findings. Unique to this study was the racially and ethnically diverse participant population enrolled from Southern California across multiple HIV risk groups, resulting in a diverse depiction of risk for HIV among cisgender women. Moreover, since cisgender women of color face social and structural barriers (e.g., stigma, medical mistrust) to uptake of PrEP use, it is critical that their experiences are represented in HIV prevention research.

### Implications for clinical practice

IPV is not only an important indicator of HIV risk and indication for PrEP, but it also may predict difficulty with adherence over time. As a result, PrEP screening and adherence support should integrate models to empower women to make feasible decisions in collaboration with their provider; this further underscores the need for the development of trauma-informed PrEP screening and adherence support tools for clinical practice. It is critical that clinicians be aware of IPV histories to best support their cisgender female patients and to customize counseling support. Given mounting data suggesting that 4 doses per week may be protective enough for cisgender women [47], clinicians may be able to tailor their adherence counseling to be more patient-centered and affirming, even in cisgender women with less than perfect adherence.

### Public health implications

Our findings suggest that screening cisgender women for IPV is vital, with a specific focus on lifetime violence. Given that lifetime physical IPV is significantly associated with decreased PrEP adherence across both weeks and months, elucidation of required levels of adherence for adequate protection from HIV infection would be beneficial. Coupling trauma services with PrEP adherence support for cisgender women at risk for HIV may help to decrease the negative impacts of trauma on physical health and decreased adherence. Clinical models that educate and empower women, such as shared decision-making and trauma-informed care, may better prepare women for understanding the importance of adherence, building self-efficacy for adherence, and executing sufficient adherence to daily oral PrEP. Further research is needed following women over time on oral PrEP, with a specific focus on violence exposure. Finally, injectable PrEP should be thoroughly explored as an alternative for cisgender women at risk for HIV who have experienced violence, given the potential for increased feasibility of adherence.

## Conclusions

Our study highlights that lifetime physical IPV is likely a significant barrier to highly adherent oral PrEP use among U.S. cisgender women. IPV can be one of many factors that act in a syndemic manner to reduce cisgender women’s initiation and adherence of PrEP as a modality for HIV prevention in high risk for HIV subgroups. Interventions to promote trauma-informed care, dedicated PrEP screening, and enhanced support for PrEP adherence among women experiencing IPV are needed.

## Data Availability

The datasets used and/or analyzed during the current study are available from the corresponding author on reasonable request.

## Abbreviations

HIV: Human immunodeficiency virus
IPV: Intimate partner violence
PrEP: Pre-exposure prophylaxis for HIV prevention
CDC: U.S. Centers for Disease Control and Prevention
AEGiS: Adherence Enhancement Guided by Individualized Texting and Drug Levels
TDF/FTC: Tenofovir disoproxil fumarate/emtricitabine
STI: Sexually transmitted infection
CASI: Computer assisted survey instrument
CTS: Conflict tactics scale
TFV-DP: Tenofovir diphosphate
LA: Los Angeles
SD: San Diego
TIC: Trauma-Informed Care
